# An Immune-Related Signature for Predicting the Prognosis of Lower-Grade Gliomas

**DOI:** 10.3389/fimmu.2020.603341

**Published:** 2020-12-08

**Authors:** Hongbo Zhang, Xuesong Li, Yuntao Li, Baodong Chen, Zhitao Zong, Liang Shen

**Affiliations:** ^1^ Department of Neurosurgery, Zhujiang Hospital, Southern Medical University, The National Key Clinical Specialty, The Engineering Technology Research Center of Education Ministry of China, Guangdong Provincial Key Laboratory on Brain Function Repair and Regeneration, Guangzhou, China; ^2^ Department of Neurosurgery, Huizhou Third People’s Hospital, Guangzhou Medical University, Huizhou, China; ^3^ Department of Neurosurgery, Peking University Shenzhen Hospital, Shenzhen, China; ^4^ Department of Neurosurgery, Jiujiang Hospital of Traditional Chinese Medicine, Jiujiang, China; ^5^ Department of Neurosurgery, The Affiliated Changzhou No. 2 People’s Hospital of Nanjing Medical University, Changzhou, China

**Keywords:** lower-grade gliomas, immune, risk score, prognostic signature, glioma

## Abstract

**Background:**

Lower-grade gliomas (LGGs) have more favorable outcomes than glioblastomas; however, LGGs often progress to process glioblastomas within a few years. Numerous studies have proven that the tumor microenvironment (TME) is correlated with the prognosis of glioma.

**Methods:**

LGG RNA-Sequencing (RNA-seq) data from The Cancer Genome Atlas (TCGA) and the Chinese Glioma Genome Atlas (CGGA) were extracted and then divided into training and testing cohorts, respectively. Immune-related differentially expressed genes (DEGs) were screened to establish a prognostic signature by a multivariate Cox proportional hazards regression model. The immune-related risk score and clinical information, such as age, sex, World Health Organization (WHO) grade, and isocitrate dehydrogenase 1 (IDH1) mutation, were used to independently validate and develop a prognostic nomogram. GO and KEGG pathway analyses to DEGs between immune-related high-risk and low-risk groups were performed.

**Results:**

Sixteen immune-related genes were screened for establishing a prognostic signature. The risk score had a negative correlation with prognosis, with an area under the receiver operating characteristic (ROC) curve of 0.941. The risk score, age, grade, and IDH1 mutation were identified as independent prognostic factors in patients with LGGs. The hazard ratios (HRs) of the high-risk score were 5.247 [95% confidence interval (CI) = 3.060–8.996] in the multivariate analysis. A prognostic nomogram of 1-, 3-, and 5-year survival was established and validated internally and externally. Go and KEGG pathway analyses implied that immune-related biological function and pathways were involved in the TME.

**Conclusion:**

The immune-related prognostic signature and the prognostic nomogram could accurately predict survival.

## Introduction

Glioma is a type of cancer that originates in glial cells, which support the nerve cells of the brain and keep the cells healthy. It is the most common primary malignant brain tumor (1). Glioma has various symptoms, including seizures, personality changes, movement difficulty, headache, problems with understanding or speaking, and vision problems. The symptoms that occur mainly depend on the tumor location as well as other tumors. According to the standards set by the World Health Organization (WHO), glioma is classified into grades I, II, III, and IV. Gliomas with histological grades II and III are identified as lower-grade gliomas (LGGs) and have highly variable clinical behaviors (2). The outcomes of LGGs are more favorable than those of grade IV gliomas. Unfortunately, the progression of LGGs occurs in almost 70% of patients within ten years (1). Aggressive high-grade gliomas have an inferior prognosis despite the treatment management with surgical resection plus radiation therapy and chemotherapy (3, 4). Because of this highly offensive ability, LGGs cannot be completely cured. Thus, delaying tumor onset and reducing tumor progression are the most challenging issues. Garcia et al. claimed that the prognosis of glioma is associated with age, sex, comorbidities, socioeconomic state, and ethnicity (5). Moreover, the study found that the absence of isocitrate dehydrogenase 1 (IDH1) mutation in LGGs was similar to glioblastoma regarding molecular and clinical characteristics (2). IDH mutation, which is considerably associated with improved prognosis, is sporadic in glioblastoma, while it is common in LGGs (6). In the analysis of the single nucleotide variation from aggregated somatic mutation of LGGs from The Cancer Genome Atlas (TCGA), we found that the mutation rate of IDH1 was 77%, and the survival rate was significantly improved in the IDH1 mutation population. Furthermore, the IDH mutation rate of CGGA was 74% with a survival protection in IDH-mutated group, which was similar to the conclusion of TCGA.

Therapeutic resistance does ultimately develop despite effective targeted therapies for tumor cells (7). Recently, research on identifying the mechanisms of resistance to therapies showed that substantial alteration occurred, not only in tumor cells but also in the tumor environment (TME). These alterations imply the importance of the extrinsic compartments of tumor cells in tumor development (8). Malignancy formation is a co-evolution of neoplastic cells together with the TME surrounded by immune cells, tumor vasculature, and extracellular matrix. The TME always dictates aberrant cellular function and affects the subsequent development of more advanced and refractory malignancies (9). Increasing evidence has extensively indicated that immune infiltrates are correlated with the prognosis of the glioma (10, 11). Indeed, immunotherapy is a novel approach utilizing the immune system against tumor progression with few short-term side effects. Thus, establishing a scientific immune-related model derived from LGG samples to predict prognosis is important.

In the current study, we screened for immune-related genes by using deep-sequencing technologies for transcriptome profiling correlated with the immune system. Univariate Cox proportional hazards regression was carried out to identify prognostic biomarkers followed by an L1 penalized least absolute shrinkage and selection operator (Lasso) Cox analysis. Multivariate Cox regression analysis was used to establish a prognostic signature to calculate the immune-related risk score that was independent of various clinical factors. A nomogram that can be utilized to personalize prognosis predictions was constructed based on age, sex, IDH1 mutation, and risk score. In addition, the prognostic signature and its independence were validated internally in TCGA and externally in Chinese Glioma Genome Atlas (CGGA). We believe that the immune-related prognostic signature will contribute to identification of potential the therapeutic biomarkers and the development of an individualized therapy guide for LGG patients.

## Material and Methods

### Patient Datasets

We extracted LGG gene information on the transcriptome in fragment per kilobase per million (FPKM) from the TCGA project (https://portal.gdc.cancer.gov/). RNA-Sequencing (RNA-Seq) data from 529 LGG tumor tissue samples and five normal brain tissue samples were screened for differentially expressed genes (DEGs). DEGs were defined as a significant difference in the expression levels of genes between in glioma and normal tissues. This procedure was implemented by R software (version 3.6.1) with the “limma” package, and we set the significance threshold as log_2_Foldchange (log_2_FC) >1 and adjusted p<0.05 for screening the DEGs with Wilcox test. The immune-related gene list was provided by the IMMPORT website (https://www.immport.org/). The intersecting gene set of DEGs and immune-related genes was used to construct the prognostic signature. The survival curve of each included gene that divided into high expression group and low expression group was mapped by R. In addition, we also downloaded the corresponding clinical information of patients from the TCGA database, including survival time, vital status, sex, age, the emergence of IDH1 mutation, and tumor grade. Samples with missing information or with a survival time less than 90 days were excluded. The dataset from TCGA was used be the training cohort, while the RNA-Seq data from the CGGA project (http://www.cgga.org.cn) was used as the testing cohort to validate the prognostic signature.

### Screening for Immune-Related Prognostic Genes and Establishing a Prognostic Signature

Univariate Cox proportional hazards regression was conducted based on the data of the training cohort for candidate genes associated with overall survival (OS). A novel algorithm, Lasso regression was applied to screen parameters in high-dimensional data (12). Lasso regression was performed on 126 genes with an adjusted p-value of less than 0.05 and further screened 25 candidate genes. Subsequently, we established a multivariate Cox proportional hazards regression model to predict prognosis based on the candidate immune-related genes. Sixteen genes with its coefficients (*β*), hazard ratios (HRs), and 95% confidence intervals (CIs) were ultimately estimated using the maximum likelihood ratio method. The risk score is a sum value that is calculated as *β* multiplied by each immune-related gene expression as follows: risk score = (expression of gene A*β_A_) + (expression of gene B*β_B_ + (expression of gene C*β_C_) +… (expression of gene N*β_N_) (13). The median risk score value of the training cohort was taken as a cutoff point for dichotomization into high- and low-risk groups (14). With the R package “survminer”, Kaplan–Meier plots and the log-rank test were used to estimate the survival rate between the low- and high-risk groups (14). A time-dependent receiver operating characteristic (ROC) curve was calculated to assess the predictive value of the multivariate Cox model (15, 16). To rule out the factors that cause accidental death in patients, such as death from postoperative complications, we excluded samples with a follow-up or OS time shorter than 90 days. The survival rate curve and ROC curve were also drawn based on the data from CGGA to validate the prognostic ability of the model.

### Independent Prognostic Role of the Immune-Related Prognostic Signature

To determine the impact of the immune-related risk score on prognosis, we need to assess whether the risk score is independent of other clinical factors, including sex, age, IDH1 mutation stage, and tumor WHO grade. Thus, univariate and multivariate Cox proportional hazards regression analyses were performed to determine the independent prognostic role of the immune-related risk score with the forward stepwise procedure. The immune-related risk score and clinical factors were deemed as independent factors if the adjusted p value was less than 0.05.

### Development and Validation of the Prognostic Nomogram

To develop an individual prognostic signature for the 1-, 3- and 5-year survival rates, a nomogram was formulated according to the significant results of the multivariate Cox proportional hazards regression model (17). We constructed this prognostic model using a backward step-down selection process with the Akaike information criterion (18). Finally, four corresponding clinical factors, including age, WHO grade, IDH mutation, and immune-related risk, were used to the develop the nomogram. The calculation of the concordance index (C-index) and the construction of a calibration curve plot were performed for the internal and external validations to check the predictive accuracy and or stability capacity of the nomogram (19). The C-index of the nomogram was observed by bootstraps with 1,000 resamples (1). The value of the C-index ranged from 0.5 to 1.0, and the size of the value determined the predictive performance of the nomogram (18). Calibration curves are used to determine the survival of the unknown sample by comparing it with the actual survival and provide a visual plot to determine the predictive of a model. A perfect calibration curve would have an R^2^ value of 1. The larger the slope of the steeper line, the more sensitive the measurement is.

### GO and KEGG Pathway Analyses of DEGs

The DEGs between immune-related high-risk and low-risk groups were screened with a log2FC >1 and adjusted p <0.05. GO analysis with functions including molecular function (MF), biological pathways (BP), cellular component (CC), and KEGG pathway analyses were performed to the DEGs by using R software at the functional level. P <0.05 and q <0.05 were considered to have a significance.

## Results

### Characteristics of the Datasets

**Figure 1:**
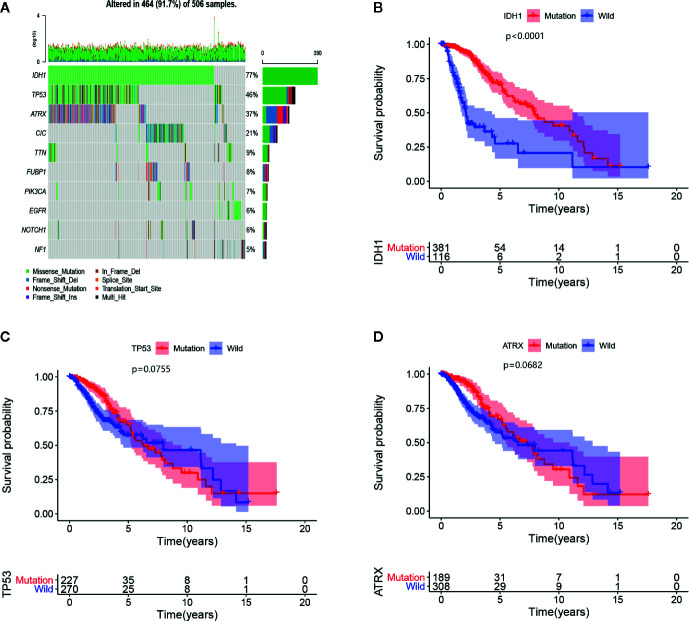
**(A)** The 10 most frequently mutated genes are displayed in the LGG samples of TCGA. The mutated rates of IDH1, TP53, and ATRX exceed 20%, and the types of IDH1 mutations are all missense mutations. **(B–D)** Survival curves of IDH1, TP53, and ATRX mutant genes, of which only IDH1 mutation affects the prognosis of LGGs.

There were 5,009 DEGs between 529 LGG samples and five normal brain tissues, 239 of which were immune-related DEGs. In the univariate Cox proportional hazards regression, 126 immune-related genes were retained for Lasso regression. Finally, 25 candidate genes were used to conduct a multivariate Cox proportional hazards regression (**Supplementary Material 1**). 459 LGG samples in training set and 362 LGG samples in the testing set with corresponding clinical information were included for the prognostic signature. In the Cox regression, we took the IDH1 mutation state into the model because we found that the most susceptible genes in LGG were IDH1, tumor protein p53 (TP53) and ATRX, chromatin remodeler (ATRX), and only the IDH1 mutation is closely associated to prognosis (**Figure 1**). The same rules were utilized to extract data from the CGGA database.

### The Immune-Related Prognostic Signature and Predictability Assessment

**Figure 2:**
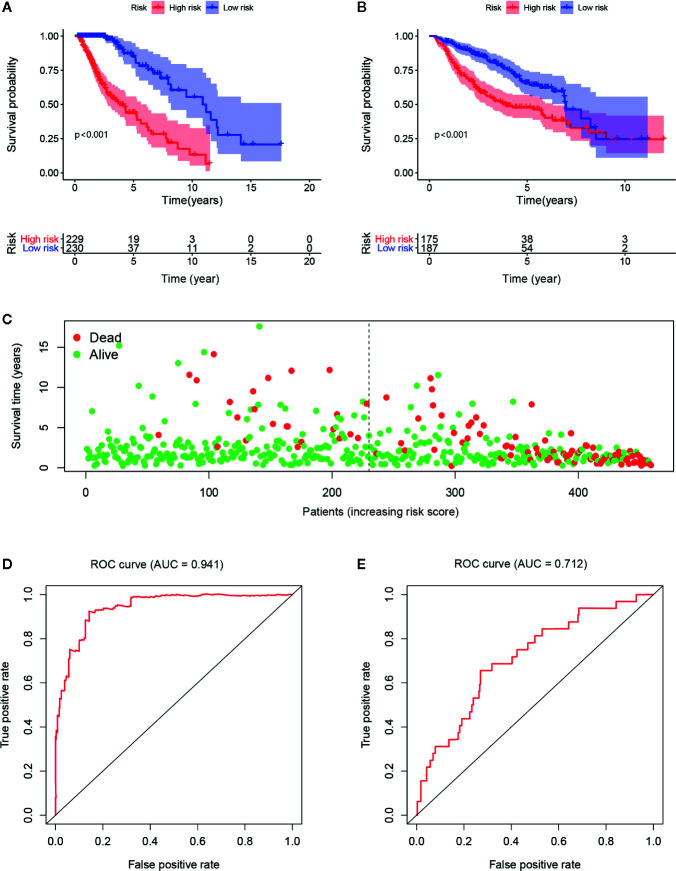
The low risk group has a better prognosis both in the training cohort **(A)** and testing cohort **(B)**. As the risk score increases, the patients’ survival rates visually decreased as well as the survival time **(C)**. The AUC values are 0.941 and 0.712 in the training cohort **(D)** and testing cohort **(E)**, respectively.

According to the relationship between the expression of significant and independent genes and OS, the risk score model with 16 immune-related genes was established by multivariate Cox proportional hazards regression. The *β* value, HR, 95CI% and p-value of each included gene in the model are shown in **Table 1**. Survival analysis revealed that 11 of the 16 immune-related genes in the signature were related to prognosis. Seven of these genes (S100A16, PLTP, IFIH1, F2R, CSRP1, APOBEC3C, SEMA5A) are considered tumor-promoting genes, and four genes (GDNF, NMB, BMPR1A, EGFR) are considered tumor-protecting genes. The immune-related risk score of each sample in the training cohort and the testing cohort was calculated in accordance with the model formula. The median risk score of the training cohort was 0.645, which was deemed as the cutoff point for dichotomizing the risk of a sample as either low- or high-risk in the training cohort (**Supplementary Material 2**) and the testing cohort (**Supplementary Material 3**). 459 LGG samples from the TCGA were divided into high-risk group with 229 samples and low-risk group with 230 samples according to the immune-related risk score. In CGGA, the samples were split into 175 samples in a high-risk group and 187 samples in a low-risk group. In addition, the risk scores of the grade II group are lower than the grade III group, as well as that in the IDH1 mutation and IDH1 wild type groups. In the training set, the low-risk patients had a much-improved prognosis (**Figures 2A, C**), and the area under ROC curve (AUC) value was 0.941 (**Figure 2D**). Moreover, a similar result was statistically significant in the testing set. Additionally, the ROC curve achieved an AUC value of 0.712 (**Figures 2B, E**). Except for FABP6, the genes incorporated into the model have a significant difference in expression between the low-risk and high-risk groups (**Supplemental Material 4**).

**Table 1:** Multivariate Cox proportional hazard regression.

Gene	*β*	HR	95%LCI	95%UCI	P value
TMSB15A	0.035	1.035	1.024	1.047	<0.001
MAVS	0.058	1.060	0.985	1.140	0.122
S100A16	0.006	1.006	1.000	1.012	0.042
FABP6	0.044	1.045	1.016	1.075	0.002
PLTP	0.004	1.004	1.002	1.006	<0.001
IFIH1	0.044	1.044	1.006	1.085	0.025
F2R	0.015	1.015	0.999	1.032	0.064
CSRP1	0.010	1.010	1.003	1.017	0.003
APOBEC3C	0.036	1.036	0.994	1.080	0.093
SEMA5A	0.049	1.050	1.033	1.066	<0.001
GDNF	−0.507	0.602	0.449	0.807	<0.001
NMB	−0.003	0.997	0.995	0.999	0.004
BMPR1A	−0.109	0.897	0.774	1.039	0.148
EGFR	0.001	1.001	1.000	1.002	0.101
BID	−0.059	0.943	0.910	0.978	0.002
CDK4	0.002	1.002	1.001	1.004	0.003

HR, hazard ratios; LCI, lower confidence intervals; UCI, upper confidence intervals; TMSB15A, thymosin beta 15a; MAVS, mitochondrial antiviral signaling protein; S100A16, S100 calcium binding protein A16; FABP6, fatty acid binding protein; PLTP, phospholipid transfer protein; IFIH1, interferon induced with helicase C domain 1; F2R, coagulation factor II thrombin receptor; CSRP1, cysteine and glycine rich protein 1; APOBEC3C, apolipoprotein B mRNA editing enzyme catalytic subunit 3C; SEMA5A, semaphorin 5A; GDNF, glial cell derived neurotrophic factor; NMB, neuromedin B; BMPR1A, bone morphogenetic protein receptor type 1A; EGFR, epidermal growth factor receptor; BID, BH3 interacting domain death agonist; CDK4, cyclin dependent kinase 4.

### Independent Predictive Role of the Immune-Related Prognostic Signature

**Figure 3:**
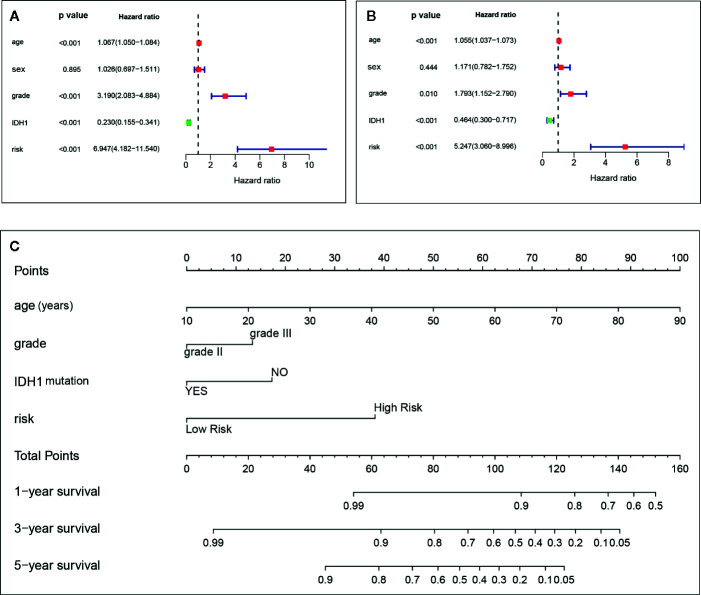
Age, grade, IDH1, and immune-related risk are independent factors in the univariate **(A)** and multivariate **(B)** Cox proportional hazards regression. The nomogram for predicting the overall survival of an individual patient. The values of age, grade, IDH1, and risk are acquired from each variable axis. The total points on the axis are the sum values of these four factors, which can predict the 1-, 3-, and 5-year survival **(C)**.

As reported before, we included the corresponding clinical information to validate the independent predictive role of the model. Sample with missing clinical information for independent prediction analysis were further excluded, and additional information can be found in **Table 2**. Univariate and multivariate Cox regression analyses were sequentially used to identify the independence of various clinical factors. Finally, the results showed that age, WHO grade, IDH1 mutation state, and the risk score calculated from the above immune-related risk score model were independent prognostic factors associated with OS. Among these independent factors, the risk score value was the most critical and played a vital role. The risk of adverse events in the high-risk group was 6.947 times that of the low-risk group in the univariate Cox regression and 5.247 times that of the low-risk group in the multivariate Cox regression (**Figures 3A, B**).

**Table 2:** Clinical information of TCGA and CGGA.

Variables	TCGA (459 samples)	CGGA (362 samples)	P value
Survival time(days)			<0.001
Median (IQR)	609(407–1120)	1031(560–1826)	
Survival State			
Alive	353	218	<0.001
Dead	106	144	
Age (years)			
Median (IQR)	41(33–53)	40(33–47)	0.001
Gender			
Female	207	157	0.621
Male	252	205	
Grade			
G2	220	159	0.253
G3	239	203	
IDH1 Mutation			
NO	102	87	0.647
YES	348	275	
NA	9	0	
High Risk			0.659
NO	230	187	
YES	229	175	

G2, WHO II; G3, WHO III; NA, not applicable; IQR, interquartile range.

### Establishing and Validating an Individualized Nomogram

**Figure 4:**
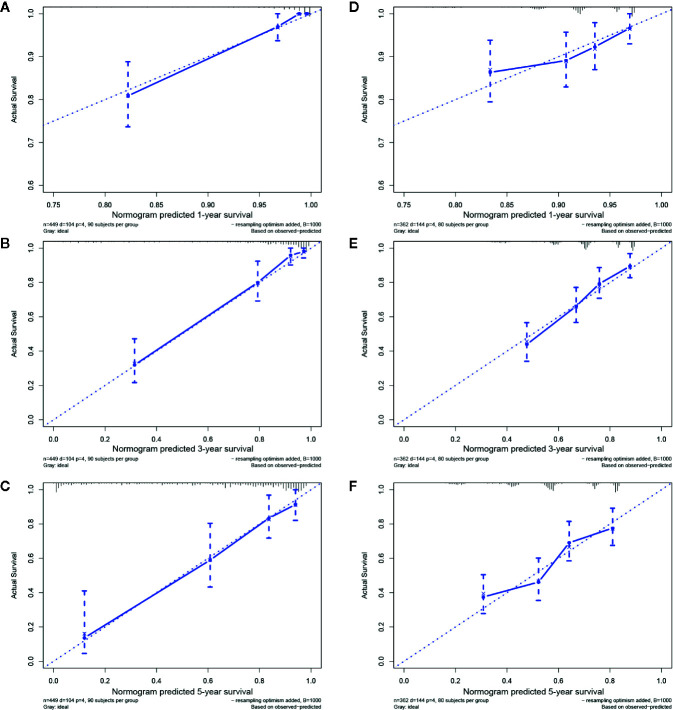
Internal and external validations in the TCGA and CGGA groups. The calibration curves for predicting 1-, 3-, and 5-year survival in the training cohort **(A–C)** and in the testing cohort **(D–F)**.

A nomogram derived from routine pretreatment parameters used in the multivariable analysis was established. The establishment of a nomogram is a crucial step in determining the likelihood of individualized predicted 1-, 3-, and 5-year survival prognoses for LGG patients (**Figure 3C**). Then, the nomogram was validated internally and externally by calculating the C-index and calibration curve, and the prediction achieved a reasonable accuracy. The C-index was 0.878 for the internal validation and 0.680 for the external validation, which indicates a consistent prediction capability. In addition, as seen from the graph in **Figure 4**, each calibration curve had goodness-off-fit.

### GO and KEGG Pathway Analyses

**Figure 5:**
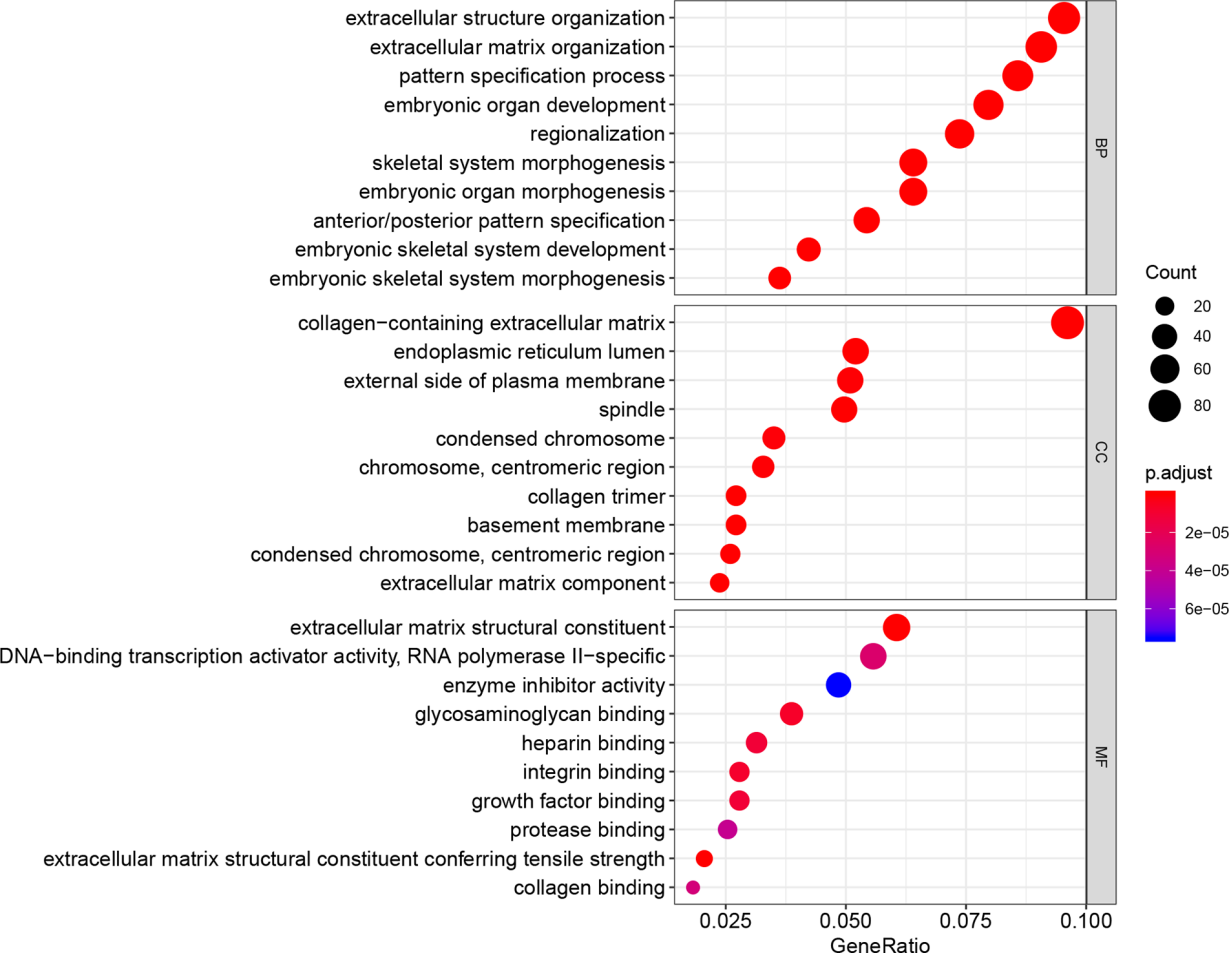
GO analysis to the 1,263 DEGs between immune-related high-risk and low-risk groups shows the top 10 listed biological function in BP, CC, and MF.

**Figure 6:**
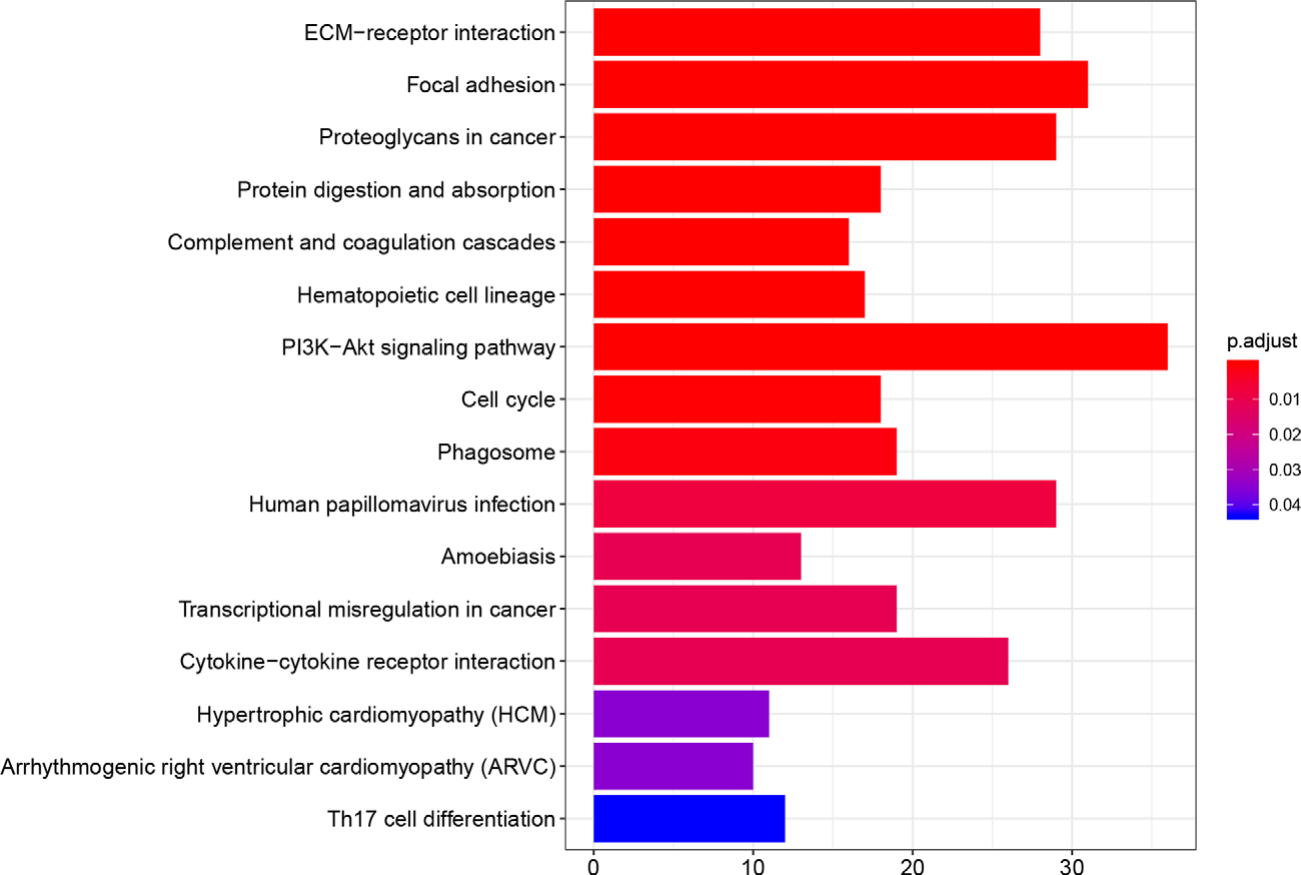
KEGG pathway analysis to the 1,263 DEGs between immune-related high-risk and low-risk groups shows the probable pathways, and some of them were immune-related.

There are 1,263 DEGs screened between immune-related high-risk group and low-risk group in TCGA. A bubble chart in the **Figure 5** shows the GO analysis of the top 10 listed based on the adjusted p value in BP, CC, and MF. GO functions, for example, extracellular matrix (ECM) function, immune response, and cytokine secretion which were related to TME in cancer were screened (**Supplemental Material 5**). KEGG analysis indicated that these DEGs were included in ECM–receptor interaction, proteoglycans in cancer, PI3K–Akt signaling pathway, cell cycle, cytokine–cytokine receptor interaction, and so on (**Figure 6**, **Supplemental Material 6**), which were related to the biological function in malignant tumors.

## Discussion

Until now, the prognosis of glioma patients varies greatly and depends on the characteristics of clinical outcomes according to the clinical practice guidelines (20). However, studies have claimed that some essential clinical characteristics, such as the WHO grade (III *vs.* IV) and resection, chemotherapy, and radiation therapy strategies, have little prognostic value (21, 22). Moreover, Parks et al. (23) did not recommend an individual prognostic model focusing on only clinical information to predict the prognosis of patients due to its imprecision. In the present study, we first screened out the immune-related DEGs in LGG. Then, 16 genes were used to establish a prognostic signature according to a multivariate Cox proportional hazards regression followed by a Lasso Cox analysis, which avoided overfitting to the greatest extent. A sixteen gene signature was identified as a prognostic signature in LGG and validated in the CGGA. Subsequently, the independent predictive role of the signature was confirmed. Finally, a personalized predicted nomogram taking risk score combined with age, IDH1 mutation, and WHO grade was formed to predict prognosis.

Clinical outcomes have been considered as the most important indicators for predicting the prognosis of malignant tumor patients. However, studies have deemed that the prognostic assessments based on clinical factors, are adequate for prognostic prediction, even for pathology classification (24, 25). However, beyond that, mutations in some favorable genes, such as IDH, TP53, and telomerase reverse transcriptase (TERT), are often used for prognostic predictions. However, the conclusion remains to be elucidated. TP53 mutations seem to have a critical effect on altering the survival time of tumor patients (26), but there was no similar effect of TP53 mutations in LGG from our survival analysis as shown in **Figure 1A**. Nonoguchi et al. (27) found that TERT mutations had a vital correlation with survival in glioblastoma, but the relationship was absent following multivariate analysis. Two years later, Simon et al. (28) suggested that TERT mutations have a predictive role in only patients with an incomplete resection and no history of temozolomide therapy. In the current study, we found that older age, WHO grade III and IDH1 mutation absence were independent factors for poor outcomes in the univariate analysis as well as in the multivariate analysis. When the patient’s age increases by one year, the unfavorable event risk increases by 5.5%. The IDH1 mutation was the only protective factor, and the risk of patients with this mutation was 0.464 times that of the patients without this mutation (**Figure 3B**). Similar results regarding older age and IDH1 mutation were put forward by Jones et al. (29). A multivariate analysis from **Figure 3B** indicated that grade III has been shown to lead to an elevated risk when compared with grade II.

As reported above, the impact of important clinical factors on the prognosis of gliomas has been known for a long time, but the influence of the gene set as a group on the prognosis of gliomas may have amazing significance. Recently, a novel approach was proposed by calculating the gene expression from RNA-Seq data, which is a far more precise measurement using next-generation sequencing technologies for transcriptome profiling than other methods (1, 13, 14, 30, 31). Studies have mainly focused on genes (30), pseudogenes (14), microRNAs (13), and lncRNAs (32) in glioma when establishing a prognostic signature. There have been some breakthrough outcomes in the treatment of gliomas, and immunological research has a pivotal position. Nevertheless, fewer studies had explored the effect of immune-related genes in a prognosis prediction model. After all, the immune system and tumor cells affect each other in prognosis (33). The immune-related risk score calculated by the prognostic signature in our study illustrates that the HR sharply increased to 5.247 in multivariate analysis. As shown in the **Figure 2D** of the ROC curve, the AUC value was 0.941, indicating that the model was accurate. The validation results from CGGA were the same as those from TCGA. Moreover, the risk score was higher in the grade III group and the IDH1 mutation group, representing a poor prognosis. Despite the lack of success of the individual prognostic calculator for glioblastoma (23), we established a prognostic nomogram for predicting the 1-, 3- and 5-years survival rate of LGG patients that was internally and externally validated and revealed that the nomogram could provide an individual prediction. The reasons why our results were different from those of Park et al. (23) could be that our subjects were patients with LGG rather than glioblastoma; moreover, another reason could be that we included the risk score in the analysis in addition to some clinical information such as age and sex. From the nomogram, we can clearly see that a high risk score accounts for a large proportion of the total points. Overall, our data lead us to the conclusion that the immune-related prognostic signature shows a powerful predictive ability in LGG.

The immune system is famous for its protection against illness and infection related to bacteria, viruses, fungi, or parasites. Interestingly, immune system is a complicated synthesis which contains stromal cells, ECM, extracellular molecules and so on, can initiate an immune response to malignant tumor. Tumorigenesis is related to the aberrant innate and adaptive immune response by selecting aggressive clones, stimulating malignant cell proliferation and metastasis, and inducing immunosuppression (34, 35). Furthermore, brain ECM was modulated in the process of glioma infiltration and it was probably a novel therapeutic target to control glioma infiltration (36). In our study, GO and KEGG pathway analyses to the DEGs between immune-related high-risk and low-risk groups implied that many biological function and pathways, for example, ECM organization, immune response, ECM–receptor interaction, cytokine–cytokine receptor interaction, and so on, probably have a significant role in immune-related tumor growth procedure. Thus, the immune system of the host in the TME plays a critical role in dictating aberrant cellular function in advanced and refractory malignancies. How do the immune system and cancer cells affect each other? The answer to this question might be explained as follows: the immune system helps to fight against cancer, while cancer can weaken the immune system, and treatments may sometimes weaken the immune system. Immune cells, including B cells, CD4 T cells, CD8 T cells, neutrophil, macrophage, and dendritic cells, are the primary functional elements in the immune system. For example, high levels of macrophage infiltration had both positive and negative correlations with tumor growth. A positive effect of macrophage infiltration on prognosis was shown in colorectal cancer, while adverse effects were displayed in breast cancer, ovarian cancer, bladder cancer, and gastric cancer (37). A high density of tumor-infiltrated T cells correlated with a good prognosis in breast cancer (38), while an elevated level of neutrophils was associated with poor outcomes in renal cell carcinoma, colorectal cancer, and glioblastoma (39). Tumor-related immune escape is achieved by avoiding immune recognition and instigating an immunosuppressive environment. The mechanism of avoiding immune recognition by cytotoxic T cells involves losing tumor antigen expression (40). On the other hand, immune tolerant is instigated by secreting suppressive molecules (41), expressing inhibitory checkpoint molecules (42, 43), and inducing the recruitment of macrophages to drive chemokines (44).

In summary, the role of the immune system in LGG has not been fully elucidated, and this study provided available information about the immune system in the tumor formation process. We believe that the prognostic signature could provide insights into predictive biomarkers or therapeutic targets for patients with LGG. Furthermore, we look forward to using the nomogram for individual prognostic assessments. However, it should be noted that the signature was established based on 16 immune-related genes and has not been proven to be the best prognostic signature. Furthermore, we used the IDH1/2 mutation for the IDH1 mutation when validating in CGGA, which may lead to an imprecise validation. However, the incidence of IDH2 mutations in LGG is scarce; it was only 3.95% in TCGA, which can be neglected when validating the model.

## Conclusions

The immune-related prognostic signature and the prognostic nomogram could accurately predict the survival.

## Author’s Note

The mechanisms of the resistance to therapies should be identified not only in tumor cells but also in the tumor environment (TME).

Prognostic signature can be used as a novel approach predicting the prognosis of patients.

## Data Availability Statement

The RNA-seq data and corresponding clinical information were observed from the TCGA (https://portal.gdc.cancer.gov/) and CGGA (http://www.cgga.org.cn). The immune-related gene list was got from the IMMPORT website (https://www.immport.org/).

## Author Contributions

All authors contributed to the study conception and design. Material preparation, data collection, and analysis were performed by HZ, XL, YL ZZ, and LS. The first draft of the manuscript was written by HZ, XL, and BC and all authors commented on previous versions of the manuscript. All authors contributed to the article and approved the submitted version.

## Funding

This work was supported by the basic research of Shenzhen science and technology plan project (No.JCYJ2017306091310788), the youth science and technology project of Changzhou (No.QN202034) and the Youth Program of Changzhou No. 2 People’s Hospital (No. 2020K001).

## Conflict of Interest

The authors declare that the research was conducted in the absence of any commercial or financial relationships that could be construed as a potential conflict of interest.

## Abbreviations

LGGs, Lower-grade gliomas; TME, the tumor microenvironment; TCGA, The Cancer Genome Atlas; CGGA, Chinese Glioma Genome; DEGs, differentially expressed genes; IDH1, isocitrate dehydrogenase 1; ROC, receiver operating characteristic; SNPs, single nucleotide polymorphisms; Lasso, least absolute shrinkage and selection operator; FPKM, fragment per kilobase per million; RNA-Seq, RNA-Sequencing; log2FC, log2Foldchange; OS, overall survival; HRs, hazard ratios; CIs, confidence intervals; C-index, concordance index; TIMER, Tumor IMmune Estimation Resource; TP53, tumor protein p53; ATRX, ATRX, chromatin remodeler; AUC, area under ROC curve; TERT, telomerase reverse transcriptase; LCIs, lower confidence intervals; UCIs, upper confidence intervals; TMSB15A, thymosin beta 15a; MAVS, mitochondrial antiviral signaling protein; S100A16, S100 calcium binding protein A16; FABP6, fatty acid binding protein; PLTP, phospholipid transfer protein; IFIH1, interferon induced with helicase C domain 1; F2R, coagulation factor II thrombin receptor; CSRP1, cysteine and glycine rich protein 1; APOBEC3C, apolipoprotein B mRNA editing enzyme catalytic subunit 3C; SEMA5A, semaphorin 5A; GDNF, glial cell derived neurotrophic factor; NMB, neuromedin B; BMPR1A, bone morphogenetic protein receptor type 1A; EGFR, epidermal growth factor receptor; BID, BH3 interacting domain death agonist; CDK4, cyclin dependent kinase 4; GO, gene ontology; ECM, extracellular matrix; not applicable; IQR, interquartile range.
