# Supplementary figures and images for: An Immune-Related Signature for Predicting the Prognosis of Lower-Grade Gliomas
Source: Front Immunol. 2020 Dec 8;11:603341. doi: 10.3389/fimmu.2020.603341 (PMC7753319; doi:10.3389/fimmu.2020.603341)

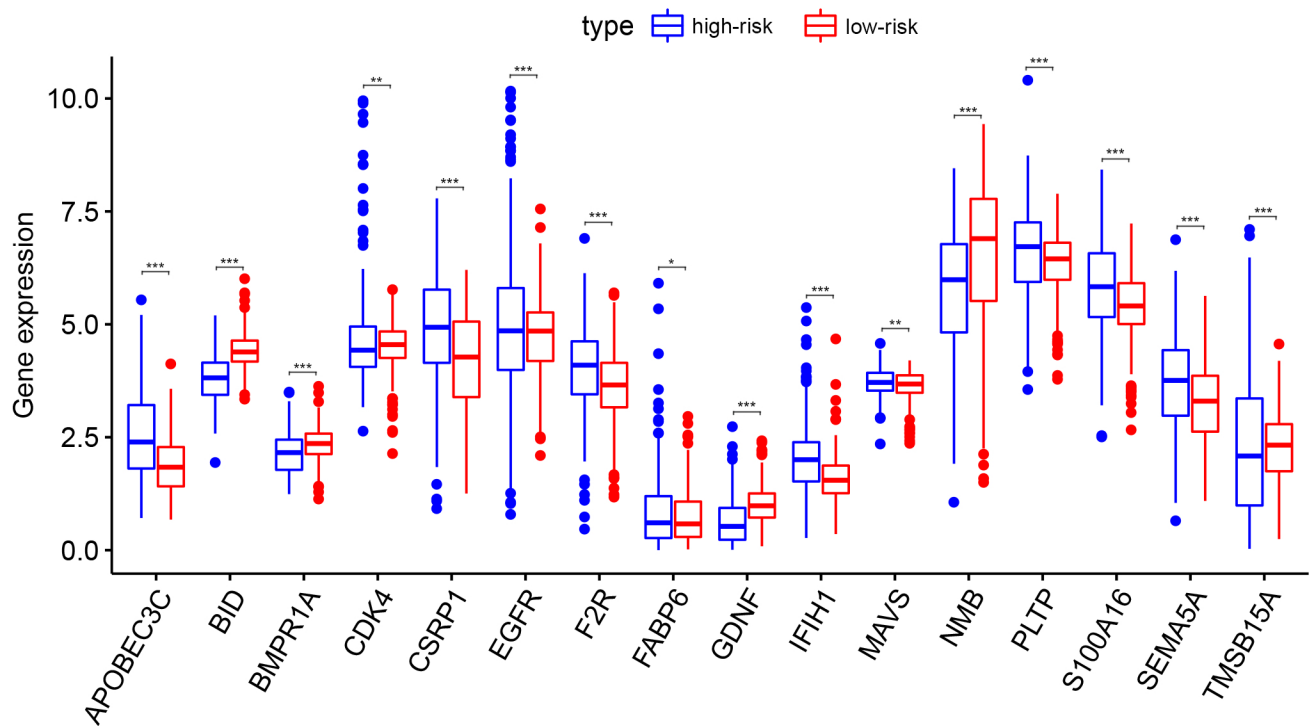

Supplement: Supplementary file 4 [file DataSheet_4.pdf]
